# Self-estimated BMI, but not self-perceived body size, accurately identifies unhealthy weight in US adults

**DOI:** 10.1186/s12889-021-10316-8

**Published:** 2021-01-30

**Authors:** Maia Phillips Smith

**Affiliations:** grid.412748.c St. George’s University School of Medicine, PO Box 7, True Blue, West Indies Grenada

**Keywords:** Self-perception, Gender, Behavior change, Obesity, Education

## Abstract

**Background:**

Self-perceptions of health and disease can be a major driver of health behaviors. Improving accuracy of self-ascertainment of obesity may prompt uptake of weight-control behaviors in those with obesity.

**Methods:**

We assess performance of self-perceived body size (‘too small’, ‘about right’ or ‘too large’), self-estimated BMI in kg/m^2^, and sociodemographics in detecting measured BMI category (under-, normal-, overweight and obese; BMI cutpoints 18.5, 25 and 30) in first bivariate and then multivariable models.

**Results:**

Of 37,281 adults in the US from NHANES, 2, 34, 33 and 32% were under-, normal-, overweight and obese. Respectively 56, 73, 60 and 91% self-perceived as ‘too small’, ‘about right’, ‘too large’ and ‘too large.’ Of those who self-perceived as ‘too small’, 22% were underweight and 10% were overweight or obese. 99.7% of obese participants self-estimated a BMI in the overweight/obese range, including many who did not self-perceive as ‘too large’.

Among obese participants, self-perception as either ‘about right’ or ‘too small’ was more likely for those who were younger (OR for perception as ‘too large’ 1.01 per year, 95% confidence interval 1.00–1.01) male (OR 0.33, (0.28–0.39)) nonwhite (ORs 0.36–0.79 for different ethnicities), low-income (ORs 0.61 and 1.8 for the lowest and highest of six categories, vs. the third) or measured recently (OR 0.98 (0.96–1.0) per year since 1999). Misperception was less common, but still existed, for participants with moderate or severe obesity (ORs 2.9 (2.3–3.5) and 7.9 (5.4–12), vs. ‘mild.’) (all *p* < 0.01.)

**Conclusions:**

A tenth of adults in the US with obesity, especially those from overweight peer groups, self-perceive as normal or underweight and thus may not be motivated to control their weight. However, virtually all self-estimate an overweight or obese BMI. If measured BMI is not available, self-estimates are sufficiently accurate that interventions may rely on it to identify obesity.

## Background

Caloric imbalance leading to excess body weight is one of the largest causes of death in the US and a major contributor to six of the top ten causes of death worldwide: [1] it is also perhaps the only behavioral risk factor which can affect individuals without their knowledge. Mortality and morbidity caused by obesity-related conditions are as severe as those caused by cigarette smoking [2]: yet unlike smokers, people with obesity may be unaware that their condition is a health risk and thus may not consider stopping the behaviors that cause it. Identifying and correcting weight misperception may increase the uptake of weight-control behaviors [3, 4] and help to mitigate the obesity epidemic.

Previous estimates of weight misperception suggest that up to half of overweight people self-perceive as healthy-weight: however, these studies tend to be based on small and/or strongly selected samples (e.g. university students, [5] physicians [6]) and thus their estimates are difficult to generalize outside these groups. However, it is known that self-perceived body size is largely based on comparison to peers [3, 7–9] and that visual adaptation to extreme body types can lead to body-size and -shape misperception even in controlled laboratory settings [10]. Thus it is not surprising that overweight individuals are less likely to self-perceive as such if they are comparable to those around them [7, 8, 11] and that in majority-overweight communities, self-perceived body size often fails to identify overweight. The same is true in the same community over time: as prevalence of overweight increases, detection of it declines [12]. However, corrected models are rare and thus it is not clear whether these effects are independent of each other: for example, the ethnic groups that are at elevated risk for misperception are also at elevated risk for overweight [8, 13]. Thus neither the prevalence nor the correlates of body-size misperception have been well established.

Furthermore, most research into size misperception does not distinguish overweight from obesity. While nonzero, the health risks of non-obese overweight are comparatively mild compared to those of obesity, [14] and these risks increase further with degree of obesity [15, 16]. Failure to ascertain obesity, especially if moderate or severe, is therefore a greater risk to health than is failure to ascertain non-obese overweight. However, neither prevalence nor correlates of misperception are well known in individuals with obesity.

Although body-size misperception is common, few interventions specifically target it; and those that do often focus on failure to ascertain underweight [17–19] rather than obesity. The assumption may be that all obese patients know their weight is outside the healthy range, or the topic may simply be an uncomfortable one: either way, many obese Americans do not know it and thus are unlikely to benefit from available tools for weight control. Physician counseling is inadequate to correct this misperception: not only are less than a quarter of Americans regularly examined by a physician [20] but physicians are often reluctant to bring a patient’s unhealthy weight to his attention [6]. Since uptake of weight-control interventions depends on initial self-perception as overweight, there is a need for an unbiased indicator of body size which can be applied by individuals to themselves.

One potential such indicator is self-estimated body mass index (BMI) which can then be categorized objectively as ‘underweight’, ‘normal weight’, or ‘overweight / obese.’ Research suggests that while self-estimation is somewhat imprecise, it is relatively accurate: one small study found an average error of 0.8 kg/m^2^ [21] while a larger one found even less [22]. Thus self-estimated BMI may be adequate to inform self-classification of weight status for most individuals. Interventions to correct misperception thus could rely on this scalable indicator, without the need for either an objective measurement or a formal physician diagnosis.

In the current study we establish the sensitivity and specificity of self-perceived body size, and self-estimated BMI, for identifying weight category and/or unhealthy weight (overweight/obesity) in a large and representative sample of adults in the US. We then establish the sociodemographic correlates of accurate self-perception as obese. In doing so we show which groups are at greatest risk for body-size misperception, in both crude and corrected models; and then evaluate whether self-estimated BMI is sufficiently accurate to serve as an alternative metric.

## Methods

### Data collection and handling

Data were collected from the National Health and Nutrition Examination (NHANES) 1999–2013, which are publicly available cross-sectional samples of adults in the US. For details on data collection and handling see the website (23)and the documentation for the relevant dataset(s).

We compared weight perception and weight estimation to objectively measured body mass index (BMI, indicated as kg/m^2^). These were quantified as follows:

- Self-estimated BMI: based on self-estimated height and weight;
- Self-perceived body size: reported as *underweight, about right,* or *overweight* [23].

Objective and estimated BMI were categorized as *underweight, normal weight, overweight* or *obese.* In adults (age 20 and up) we used the BMI cutpoints published for adults by the US CDC: 18.5, 25, and 30 kg/m^2^ [24]. In adolescents (age 16–19) we used age- and sex-specific 5th, 85th, and 95th BMI percentiles of a reference population of Americans [25] as recommended by the CDC [26].

For maximum comparability between self-perception and self-estimation, some analyses combined self-estimated overweight and obesity into a single category corresponding with self-perceived ‘overweight.’

In some analyses, obesity was subset into class 1, class 2, or class 3 based on BMI cutpoints of 35 and 40 as recommended by the CDC [26].

As variables associated with obesity and/or weight misperception we considered age;

sex; year of followup; ethnicity; objectively measured BMI as percent of self-estimated; and household income.

### Statistical methods and modeling

All analyses were performed using SAS University Edition. Statistical significance was set at *p* = 0.05 unless otherwise stated. Analyses were limited to participants with complete data on measured BMI, on estimated BMI, and on perceived body size. Because perceived body size was collected only for participants over 15 years of age, all analyses were limited to this age group.

All group comparisons were done using survey-weighted logistic regression. Sample weights, including stratification and clustering, for descriptive and analytic models were taken from NHANES. Sample weights were those for participants who underwent a physical exam at the Mobile Examination Center (WTMEC2YR.)

We first established prevalence and correlates of the four weight categories (underweight, normal weight, overweight, obese) in our population and established sensitivity and specificity of self-perception and self-estimation for ascertaining objectively-measured BMI category (the gold standard). Because the provided measure of self-perceived body size did not distinguish overweight from obesity, we repeated the previous analyses treating self-estimated overweight or obesity (equivalent to adult BMI over 25) as a single category. This maximizes comparability of self-perception with self-estimation. Using logistic regression, we then modeled associations of each risk factor with accuracy of weight perception in obese participants. In these models, the outcome was self-perception as ‘too large.’ Predictors that were bivariate statistically significant then entered mutually corrected models and were removed stepwise in order of significance.

## Results

### Baseline prevalences and correlates

Prevalences of underweight, normal weight, overweight and obesity were 1.92, 33.5%, 32.5 and 32.1% respectively. (Table 1.) Of obese participants, 59% had class 1 obesity; 25% had class 2; and 17% had class 3. Most group comparisons were statistically significant at *p* < 0.01, so we state only *p*-values that were less extreme than this.

**Table 1:** Population Profile. Non-pregnant participants ages 16 and up in NHANES 1999–2013, complete data on BMI and weight perception. *N* = 37,281

	Underweight	Normal weight	Overweight	Obese	P-value for difference from normal weight	
					Underweight	Obese
Percent of total	1.9	34	33	32	–	–
Date measured (years since 1999)	6.9	7.0	7.6	8.0	> 0.10	***
Age, years	38	40	47	46	0.03	***
Male	36	44	58	47	***	0.001
Ethnicity	–	–	–	–	0.01	***
African-American	11	9.2	9.9	15	> 0.10	***
European-American	71	70	70	67	(ref)	(ref)
Hispanic	7.5	12	15	14	0.02	***
Other	11	9.0	5.4	3.9	> 0.10	***
Household income as % of FMPI	–	–	–	–	***	***
< 100	24	15	13	15	0.002	0.02
100–200	25	19	19	22	0.04	> 0.10
200–300	13	14	15	16	(ref)	(ref)
300–400	14	14	14	14	> 0.10	> 0.10
400–500	9.0	10	11	11	> 0.10	> 0.10
> 500	15	27	28	21	0.01	***
Obesity class					n/a	n/a
1				59	–	–
2	0	0	0	25	–	–
3				17	–	–
Perceived body size	–	–	–	–	n/a	n/a
Too small	**56**	10	1.0	0.54	–	–
About right	43	**73**	39	8.6	–	–
Too large	1.3	17	**60**	**91**	–	–
Self-estimated weight category based on BMI	–	–	–	–	n/a	n/a
Underweight	**67**	1.2	0.02	0.008	–	–
Normal weight	33	**92**	14	0.25	–	–
Overweight	0	6.6	**81**	16	–	–
Obese	0	0.14	4.3	**84**		
Measured BMI as % of self-estimated: mean, 5th, 95th percentiles	97 87, 106	100 92, 108	102 95, 111	104 96, 116	***	***

Continuous measures expressed as mean unless stated otherwise. Binary measures expressed as %

All p-values from survey-weighted logistic regression, with a binary outcome. P-value for top row of ethnicity, BMI category, and income is type 3 test of null hypothesis (all categories equal)

*P*-value for each row of categorical measures is that for pairwise comparison with normal weight

*** if *p* < 0.0001, n/a if not tested

1) *P*-value that people with overweight, obesity, or both groups differ from normal weight

Obesity was more common in more recent years (mean years since 1999 were 8.00 for obese participants, vs. 7.63 and 6.99 for overweight and normal weight). Overweight participants were older, and more likely to be male, than either obese or normal-weight participants (46.7 years, vs. 46.0 and 39.9; 58% vs. 44 and 47%.)

Self-perceived body size matched objectively measured BMI category for 56% of participants with underweight, 73% of those with normal weight, 60% of those with overweight, and 91% of those with obesity. Objectively-measured BMI averaged 97, 100, 102 and 104% of self-estimated for each group, and 90% of each group estimated their BMI within about 5% of the group mean on either side. 86% of participants with overweight estimated a BMI within the overweight/obese range, as did 99.7% of those with obesity.

Errors in self-perception were usually, but not always, between adjacent categories. One percent of participants with measured overweight, and 0.54% of those with measured obesity, self-perceived as ‘underweight.’ Of those with measured underweight, 1.3% self-perceived as “too large”; this corresponds to about 24 per 100,000 population.

### Sensitivity and specificity of perceived body size

Self-perception as ‘too large’ had a sensitivity of 60% in detecting measured overweight and 91% in detecting measured obesity. (Table 2.) Its specificity was 90% in detecting combined overweight and obesity.

**Table 2:** Test Characteristics. Non-pregnant participants ages 16 and up in NHANES 1999–2013, complete data on BMI and weight perception. N = 37,281

		Objectively-measured BMI category			
		Underweight	Normal weight	Overweight	Obese
Self-perceived body size	Sensitivity	55.7	73.2	59.6	90.8
	Specificity	21.7	59.9	89.6	
BMI category based on self-reported height and weight	Sensitivity	67.1	92.1	81.4	83.5
				99.7	
	Specificity	76.2	85.2	78.1	94.8
				96.4	

Sensitivity and specificity refer to the ability of individual self-perception or self-estimation to identify objectively measured BMI category. Self-perceived body size was categorized into ‘underweight’, ‘about the right weight’ or ‘overweight’, with no separate category for obesity

Self-perception as ‘underweight’ had a sensitivity of 56% in detecting measured underweight, and a specificity of 22%.

### Sensitivity and specificity of estimated BMI

Estimated BMI had a sensitivity of 81% for detecting overweight, and 84% for detecting obesity. Its specificity was 78 and 95%, respectively. When overweight and obesity were combined in a single group, that group was detected by estimated BMI with a sensitivity and specificity of 93 and 96%.

Estimated BMI had a sensitivity of 67% in detecting measured underweight, and a specificity of 76%.

### Detection of obesity

In corrected models predicting accurate self-perception of obesity (Table 3) most sociodemographic predictors remained statistically significant after correction for each other. Female gender was a significant predictor of accurate perception, as was high income and European-American ethnicity. All *p*-values were < 0.001 unless stated otherwise.

**Table 3:** Correlates of Obesity Perception. Non-pregnant participants ages 16 and up in NHANES 1999–2013, complete data on BMI and weight perception; BMI ≥ 30 or above 95th percentile

	Accurate (self-perceives as ‘too heavy’)	Inaccurate (self-perceives as ‘about right’ or ‘too thin’)	Odds ratio predicting accuracy^1^			P
			Point estimate	95% confidence interval		
				Bottom	Top	
Percent of total obese	9.1	91	–	–	–	–
Years since 1999	8.0 0, 13	8.5 0, 13	0.979	0.962	0.996	0.02
Age, years	46 20, 73	43 17, 76	1.01	1.00	1.01	0.003
Male	45	68	0.326	0.277	0.385	***
Ethnicity	–	–	–	–	–	***
African-American	14	26	0.356	0.299	0.425	***
European-American (ref)	69	50	1.0	1.0	1.0	(ref)
Hispanic	14	20	0.730	0.599	0.890	0.002
Other	3.8	4.2	0.791	0.529	1.18	> 0.10
Household income as % of FMPI	–	–	–	–	–	–
< 100	14	24	0.608	0.472	0.783	0.0001
100–200	22	28	0.734	0.574	0.940	0.01
200–300	16	17	1.0	1.0	1.0	(ref)
300–400	15	12	1.31	0.956	1.79	0.09
400–500	11	5.3	2.21	1.48	3.31	0.0001
> 500	22	14	1.83	1.34	2.49	0.0001
Obesity class	–	–	–	–	–	–
1 (ref)	56	83	1.0	1.0	1.0	(ref)
2	26	14	2.86	2.33	3.52	***
3+	18	3.5	7.92	5.35	11.7	***
Self-estimated BMI as % of measured	104 95, 115	105 95, 120	n/a	n/a	n/a	n/a
Category of self-estimated BMI	–	–				
Underweight	0.002	0.060				
Normal weight	0.17	0.99	n/a	n/a	n/a	n/a
Overweight	15	33				
Obese	85	66				

Continuous measures expressed as mean; 5th, 95th percentiles. Binary measures expressed as %

*P*-value for top row of ethnicity, BMI category, and income is type 3 test of null hypothesis (all categories equal.) *P*-value for each row is that for pairwise comparison with the reference group. *** if *p* < 0.0001, n/a if not tested

1) Survey-weighted logistic regression used to fit multivariable corrected models

Obese participants were less likely to self-perceive as ‘too large’ if they were followed up more recently (OR for accurate perception 0.979 per year) or if they were older (OR 1.01 per year) or male (OR 0.326.) There was also a clear trend of increasing accuracy in weight perception across income categories: obese people earning below the poverty line had an OR of 0.608 for detection, while the next categories had ORs of 0.734, 1 (reference), 1.31, 2.21, and 1.83 (all pairwise *p*-values ≤0.05.)

All ethnic groups were at higher risk of misperception than was the European-American reference group: odds ratios were 0.356, 0.730, and 0.791 (pairwise *p* > 0.10) for African-American, Hispanic, and ‘other race’ respectively.

Lastly, obesity class had an effect: 83, 14, and 3.5% of obese participants who self-perceived as ‘about the right size’ were class 1, 2 and 3 respectively, compared with 56, 26 and 18% of those who self-perceived as ‘too large’ Corrected odds ratios for accurate perception were 2.86 for class 2 and 7.92 for class 3, compared to class 1 (reference.)

## Discussion

We find that even in the presence of body-size misperception, self-estimated BMI can accurately identify obesity. 9% of participants with obesity, including some with moderate or severe obesity, self-perceived themselves as being either the right weight or underweight. However, over 99% estimated a BMI within the overweight/obese range, which suggests that self-estimated BMI can adequately identify obesity.

Our study confirms some previously-found correlates of weight misperception among overweight/obese individuals, [9] such as Black and Hispanic ethnicity; and identifies some new ones, such as male gender. The increased risk of misperception among those followed up more recently supports previous findings of a generational effect in overweight perception [12]: we suggest this may be due to the tendency of individuals to base their eating habits [11] and perceptions of body size [8, 27] on the people around them rather than any objective standard. This is further supported by our observations, and those of others, [9] that obesity misperception was often more pronounced in groups where obesity was more prevalent. To this observation we add that the effects of group membership were almost independent: the elevated risk of misperception among ethnic minorities remained even after correction for income.

Failure to ascertain obesity was more common in males, those with low income, and those from ethnic minority groups. These groups are at relatively low risk for disorders associated with the failure to ascertain underweight, such as anorexia nervosa [17] but at high risk for low health literacy [28, 29] and its consequences. This suggests that accuracy of ascertainment of obesity may be an aspect of health literacy rather than a symptom of disordered eating per se. This supports the findings of Bullivant et al., who showed that although obesity has similar correlates and symptoms to eating disorders (EDs) it is considered to be a separate condition by both health practitioners [30] and members of the public [31]. Given the association between low health literacy and obesity-related conditions such as heart failure, [28] it is plausible that interventions shown to improve other aspects of health literacy could be adapted to encourage accurate self-perception of body size. Such interventions may include the use of self-estimated BMI over 25 as a scalable, sensitive and specific size indicator.

Inaccuracies in estimation of BMI contributed to inaccurate classification mostly in those participants whose BMI was relatively close to the cutoff. These included many participants with underweight, normal weight and non-obese overweight, but almost none with obesity: almost all participants with obesity self-estimated their BMI as being over 25. Thus while there are systematic errors in self-estimated BMI, these errors have only minimal effects on BMI-based ascertainment of obesity.

Lastly, while this paper focused on self-perception in individuals with obesity it also informs practice pertaining to underweight. Over three-fourths of participants who perceived themselves as underweight were incorrect, and about 10% of them were in fact overweight or obese. In the absence of objective measures, almost all perceived underweight is incorrect: treatment should not begin until the perception is confirmed by objective measures [17, 19].

### Study limitations

This study is limited, first of all, by its cross-sectional observational nature. While research does support self-perception of body size as a major driver of the decision to lose weight, it is not the only driver.

We are also limited by the lack of data on Asian origin for most participants [32]. The BMI cutpoints we used to ascertain weight categories were chosen for European-origin populations in spite of the known between Europeans and different populations of Asians [33]; thus we likely misclassified some individuals of Asian origin whose BMI was near the cutoff. Consequently, our findings for individuals of ‘other ethnicity’ (which includes all Asians) may be somewhat less reliable than those for the other ethnicities [32, 33].

## Conclusions

These findings suggest that screening and treatment decisions for abnormal body weight (both over- and under-) should rely on BMI rather than subjective perception. Objective measures are best, but self-estimated BMI over 25 is also an adequate screening tool for obesity. Clinicians should consciously inform their subjective perception of body size to match the BMI cutoffs. Ethnic minorities, low-income people, individuals from majority-overweight communities, and men are least likely to be aware of their own obesity and thus, if properly counseled, may be more willing to lose weight.

## Data Availability

Publicly available at https://wwwn.cdc.gov/nchs/nhanes/search/default.aspx

## Abbreviations

BMI: Body mass index, kg/m^2^
CDC: US Centers for Disease Control
FMPI: Ratio of household income to poverty limit
